# Editorial: Sexuality 3.0

**DOI:** 10.3389/fsoc.2022.1106569

**Published:** 2023-01-04

**Authors:** Stefano Eleuteri, Valeria Saladino

**Affiliations:** ^1^Department of Psychology, Sapienza University of Rome, Rome, Italy; ^2^Department of Human Sciences, Society and Health, University of Cassino and Southern Lazio of Cassino, Cassino, Italy

**Keywords:** sexuality, attachment style, intimate relationships, gender, COVID-19

Sexuality is a part of the human being. To date, human relationships have changed, involving more affective and sexual issues associated with difficulties of intimacy, such as interpersonal violence within the couple. Our Research Topic aimed to analyze the new forms of sexuality and relationships associated with social networks, gender fluidity, sexual dysfunctions, interpersonal violence, and the influence of COVID-19 on sexuality and sexual identity.

In this regard, eight articles were included in our special issue, covering the main themes of the topic. van Lankveld et al. offer an overview of the associations between relational needs related to attachment, partner reactivity, intimacy, and sexual desire. The authors assume that the emotional intimacy and perceived reactivity of the partner vary according to their needs related to attachment and influence sexual motivation, consciously experienced as sexual desire. They conducted a study on dyadic sexual desire in individuals involved in a committed romantic relationship. The results show that attachment style, partner responsiveness, and emotional intimacy positively correlate with and influence sexual desire. Individuals with anxious and avoidant attachment styles are characterized by anxiety and insecurity, but the difference is derived from the coping strategies implemented by the participants. Indeed, those with anxious attachment amplify their anxiety and seek support from their partner, translating into a strong sexual desire. Instead, individuals with avoidant attachment try to manage their anxiety by creating distance from the other, thus manifesting a very poor level of sexuality.

Related to attachment style, the role of sex in intimate relationships is a topic of relevant interest (Zhang). There are two fundamental intersubjective relationships: “I-Thou” and “I-It”. The first requires that the subject can understand the differences with others and interacts with the partner with sincerity (Morgan and Guilherme, 2013). In this type of relationship, both members of the couple fully participate in the balanced combination of sexual desire and emotion. However, in some difficult situations, sexual intercourse can break down. On the other hand, in relationships dominated by the “I-It” mode, the two parties can often not be considered equal. In such relationships, sex reflects problems of intimacy, deception, or violence and becomes a power struggle between the two partners, who can implement different strategies of interaction. One of these strategies is *control*, used to obtain sexual pleasure, by ignoring the partner's emotional needs. Another strategy implemented in this type of relationship is *compliance or submission*. At last, a third strategy is *avoidance*, characterized by individuals who end an affective relationship, showing indifference, and self-sufficiency in sexual behavior (Alperin, 2001). These individuals are often resistant and fearful of intimacy. As a result, people who adopt this strategy could tend to use pornography and masturbation.

Gender and fluidity are defined by the inactive-ecological approach (Albarracin and Poirier). According to this approach, individuals perceive the world as a field of affordance, that is, as structured sets of possibilities of action. Previously, gender was often interpreted as a static binary state that people personified according to the assigned biological sex (Björkqvist and Österman, 2018). From a cultural perspective, however, gender could be interpreted as dynamic (Coney, 2015). Similarly, new identification possibilities that mix male and female components have become possible. People who identify themselves as no-binary, aliagender, aporagender, or agender have completely moved out of this conceptual boundary (McCarthy et al., 2022). Although they aren't yet entirely mainstream, these identifications are being adopted more and more, mainly within the younger population, which has been less exposed to more traditional gender roles (Richards et al., 2016). These gender identifications allow for new script clusters. Some LGBT individuals often renounce to their sexual identity to maintain their religiosity beliefs. For instance, gay Muslims choose to marry a woman, contributing to the phenomenon of Gay Men in Straight Marriages (GMiSM). In this regard, Zulkffli et al. conducted a case study with two homosexual and Muslim men from Malaysia, Fahrin, and Muzz. This research confirmed the prevalence of infidelity among GMiSM and highlighted the misogyny perpetuated by these subjects toward their wives. Since gay men in Malaysia are marginalized, the position of men allows them to oppress women. Since the Islamic identity is an integral part of Malaysian identity, capturing the entanglement of the participants' “illegitimate” sexuality and the consequent infidelity with the beliefs of their faith through their spoken speech would be crucial to providing a new vision of the GMiSm phenomenon in the Malaysian-Muslim context. Through discursive psychology and the discursive action model (Edwards and Potter, 1992), the authors explain how Muzz interpreted through his religiosity the event that led to his repentance and how he worked to support Malaysian-Muslim heteronormative hegemony in Malaysia. The LGB theme was enriched in the special issue by the article by Wang, Liu et al. on the validation of Herek's (1988) attitudes scale in China. The authors highlighted that there was still a lack of a standardized reliable and valid instrument to measure attitudes toward lesbian women and gay men in China, posing a challenge to compare and contrast intervention measures.

Sexual behaviors and predictors remain one of the most important aspects in sexuality research, suggesting further needs for implementing sexual education. Wang, Jin et al., on the basis of the theory of planned behavior (TPB), conducted an investigation of the factors influencing the sexual behavioral intentions of Chinese college students. Their analysis revealed the following: (1) subjective norms and behavior control are key variables that influence the safe sexual behavior of college students; (2) attitudes and safe-sex behavioral intentions are influential in groups with extensive sexual knowledge; (3) behavioral control and subjective norms influence the differences in the comparative sexual knowledge of students. Luz et al. focused on casual sexual relationships, underlying the ambiguities that still emerged from the focus group interviews and the need to better prepare and empower young adults in their sexual and relational trajectories. Finally, Eleuteri et al. discussed in their review how the COVID-19 pandemic and its related restrictions significantly impacted individuals' health, wellbeing, and security. According to the main contents, the importance of the bio-psycho-social approach is underlined, considering cultural changes in the sexological context.

## Author contributions

SE wrote the first draft of the manuscript. VS wrote the final draft. Both authors have reviewed, discussed, and accepted the final version.
